# A systematic review of multicomponent vs. single-component training programs for fall prevention in older adults

**DOI:** 10.3389/fpubh.2025.1636439

**Published:** 2025-07-28

**Authors:** Krzysztof Kasicki, Ewa Klimek Piskorz, Łukasz Rydzik, Tadeusz Ambroży, Piotr Ceranowicz, Wiesław Błach

**Affiliations:** ^1^Department of Physiotherapy, Faculty of Health Sciences, Collegium Medicum, Andrzej Frycz-Modrzejewski Krakow University, Kraków, Poland; ^2^Department of Rehabilitation in Rheumatology and Geriatrics, Institute of Clinical Rehabilitation, Faculty of Physical Rehabilitation, University of Physical Culture, Kraków, Poland; ^3^Institute of Sports Sciences, University of Physical Culture, Kraków, Poland; ^4^Department of Physiology, Faculty of Medicine, Jagiellonian University Medical College, Kraków, Poland; ^5^Faculty of Sport, University School of Physical Education in Wroclaw, Wrocław, Poland

**Keywords:** fall risk, older adults, training programs, functional training, functional parameters

## Abstract

**Background:**

Falls among adults aged 60 years and older often result in serious injury, yet studies evaluating single-component exercise interventions have not been directly compared with those incorporating multiple modalities. This review set out to assess whether multicomponentl training programs combining strength, balance, and aerobic exercises are more effective at preventing falls in older adults than single-component regimens.

**Methods:**

A systematic search of PubMed, Scopus, Web of Science and EBSCO yielded 284 records, of which six randomized controlled trials (*n* = 40–670; age range 60–80 years) met inclusion criteria. All interventions lasted at least 6 weeks. We narratively synthesized data on functional outcomes (Timed Up and Go [TUG], Star Excursion Bal-ance Test [SEBT], 30-Second Chair Stand [CS-30], gait speed), physiological measures (VO₂max, lean body mass, bone mineral density) and fall incidence rate ratios (IRR).

**Results:**

Multicomponent programs surpassed single-component interventions, producing a ~ 1-s faster TUG, an 8–20% improvement in SEBT reach, and a 55% increase in CS-30 repetitions. Physiologically, VO₂max rose by 154 mL/min, lean body mass by 1.2 kg, and BMD by 0.02 g/cm^2^. The greatest reduction in fall risk was observed in the Tai Ji Quan group (IRR 0.42), compared with 0.60 in the standard multicomponent program.

**Conclusion:**

Protocol heterogeneity precluded quantitative meta-analysis. Based on limited and heterogeneous data from six RCTs, MULTI programs appear to improve selected functional parameters and reduce fall risk; however, the effectiveness of the single-component UNI program (Tai Ji Quan) was comparable to—or even greater than—that of MULTI in reducing actual falls.

**Systematic review registration:**

https://www.crd.york.ac.uk/PROSPERO/view/CRD420251045931.

## 1 Introduction

Falls represent a significant threat to individual health. Their consequences may include bone fractures, ligament ruptures—collectively referred to as musculoskeletal injuries—but are not limited to these outcomes (1). Approximately 20% of falls in older adults result in serious injury (1). In some cases, the outcome is death, following severe health deterioration caused by the fall. As reported by researchers, the percentage of fall-related deaths has been systematically increasing each year (2). This trend correlates with the rapidly growing proportion of older individuals in highly developed countries, particularly those located in Europe (3, 4). In Europe, by 2050, the proportion of people over the age of 65 is projected to exceed 30% of the total population, which will translate into a dramatic rise in fall incidents (5).

The complexity of the issue stems from the multidimensional nature of fall risk, which can be divided into intrinsic factors (related to individual health status) and extrinsic factors (environmental) (6). Among intrinsic factors, key roles are played by muscle weakness (sarcopenia), impaired balance and coordination, reduced joint flexibility, di-minished proprioception, neurological conditions (e.g., Parkinson’s disease), cardiovascular issues (e.g., syncope, orthostatic hypotension), and visual impairments (7–10). Additionally, polypharmacy increases the risk of cognitive and balance disorders, posing an added threat (11). In terms of extrinsic factors, hazardous architectural barriers, inappropriate footwear, slippery surfaces, and poor lighting should be highlighted (12).

Over the years, numerous studies have promoted methods proven to reduce fall risk (13–17). Most can be categorized as either single-component approaches—focusing on one type of activity (e.g., strength training, balance exercises, aerobic classes)—or multicomponent interventions that integrate various exercise modalities, often combined with educational elements, environmental modifications, or medication reviews (18–22). Single-component programs typically target isolated improvement of one physical “ability.” For example, resistance exercises aim to strengthen the lower limbs, while balance training might include exercises on unstable surfaces. Their advantages lie in protocol clarity and the ability to precisely measure intervention outcomes; however, they lack a comprehensive approach to all fall-related risk factors (23). Additionally, participants often be-come quickly bored with monotonous programs, which may reduce motivation and adherence.

Multicomponent programs, on the other hand, combine various exercise forms—strength, balance, flexibility, coordination—and sometimes cognitive training or environmental adaptations within a single protocol. This allows them to address multiple risk factors simultaneously (24). However, although such approaches integrate many motor skills at once, the development of specific components such as coordination or strength requires longer time investment and proper periodization (25, 26). According to fundamental principles of training theory and physiological adaptation, focusing on a single motor ability may yield stronger effects (27, 28). With multi-stimulus training, this specificity is not fully achievable. This presents a challenge for clinicians when designing and prescribing training or preventive units for older individuals at risk of falling.

In light of the growing number of studies on fall prevention in older adults, most have focused on individual training components—strength, balance, or aerobic capacity—demonstrating their isolated benefits. However, such studies do not fully reflect the complexity of risk factors. While single-component protocols are easy to standardize and evaluate, their limited scope may reduce their clinical effectiveness, especially considering the co-occurrence and mutual reinforcement of risk factors such as sarcopenia, balance impairments, and structural changes (29–32). Conversely, multicomponent programs theoretically address a wide spectrum of deficits by combining strength, balance, and flexibility training, sometimes with behavioral or educational components. However, a comprehensive synthesis and comparison of their effectiveness relative to single-component interventions in fall prevention has so far been lacking. Previous reviews have focused solely on the effectiveness of individual modalities (e.g., balance, strength), rarely comparing UNI and MULTI programs directly (33, 34). Our review addresses this gap by simultaneously analyzing both functional parameters and actual falls.

Therefore, the aims of this review are to:

- Compare the effectiveness of multicomponent (MULTI) and single-component (UNI) programs in fall prevention among older adults, considering both functional outcomes (balance, strength, mobility) and fall incidence rates (IRR).
- Identify which intervention components (type, dosage, duration) and proto-col characteristics (periodization, educational support) are associated with the greatest health benefits.
- Provide practical recommendations for clinicians on how to optimally select and design training interventions for comprehensive fall risk reduction in community-dwelling older adults.

## 2 Methods

This systematic review was conducted in accordance with the Preferred Reporting Items for Systematic Reviews and Meta-Analyses (PRISMA) guidelines and was prospectively registered in the PROSPERO database under the ID: CRD420251045931.

### 2.1 Literature search strategy

To identify and narrow down the number of publications relevant to the topic, search strategies incorporating specific keywords were developed. The following databases were searched: PubMed, Scopus, Web of Science, and EBSCO, with no time restriction on the publication date up to April 1, 2025. After applying the search strategies, filters were used as follows: “randomized controlled trials” in PubMed and “English language” in all data-bases. The search formulas are presented below:

- PubMed:

((“circuit training” OR circuit-based OR multicomponent OR multimodal OR “combined exercise”) AND (“balance training” OR “isolated balance” OR “postural control” OR “postural stability”) AND (falls OR “fall prevention” OR “fall risk” OR “accidental falls”[Mesh]) AND (“older adults” OR older adults OR aged OR geriatric OR senior OR “community-dwelling”)).

- Scopus:

TITLE-ABS-KEY ((“circuit training” OR circuit-based OR multicomponent OR multimodal OR “combined exercise”) AND (“balance training” OR “isolated balance” OR “postural control” OR “postural stability”) AND (falls OR “fall prevention” OR “fall risk” OR “accidental falls”) AND (“older adults” OR older adults OR aged OR geriatric OR senior OR “community-dwelling”)).

- EBSCO:

((“circuit training” OR circuit-based OR multicomponent OR multimodal OR “combined exercise”) AND (“balance training” OR “isolated balance” OR “postural control” OR “postural stability”) AND (falls OR “fall prevention” OR “fall risk” OR “accidental falls”) AND (“older adults” OR older adults OR aged OR geriatric OR senior OR “community-dwelling”)).

- Web of Science:

TS = ((“circuit training” OR circuit-based OR multicomponent OR multimodal OR “combined exercise”) AND (“balance training” OR “isolated balance” OR “postural control” OR “postural stability”) AND (falls OR “fall prevention” OR “fall risk” OR “accidental falls”) AND (“older adults” OR older adults OR aged OR geriatric OR senior OR “community-dwelling”)).

### 2.2 Eligibility criteria

To effectively identify publications of the highest relevance to the review topic, the PICOs strategy was employed to establish the eligibility criteria for article selection. Only randomized controlled trials were included in the analysis, with intervention durations of 6 weeks or longer. Studies were required to report quantitative functional outcomes to be considered for evaluation. Case–control and pilot studies were excluded. Gray literature and non-English language studies were excluded from this review due to limited resources for independent translation and existing evidence indicating a low prevalence of high-quality RCTs in such sources. This limitation is acknowledged as a potential source of bias. The detailed inclusion and exclusion criteria are presented in Table 1.

**Table 1:** Inclusion and exclusion criteria based on the PICOS framework.

PICOs model	Inclusion criteria	Exclusion criteria
Population	Older adults, aged ≥ 60 years	Hospitalized individuals; Individuals with exacerbations of chronic diseases
Intervention	Multicomponent programs (“MULTI”), defined as interventions including ≥ 2 training components: Strength training (progressive resistance, bodyweight exercises) Balance exercises (static and dynamic) Aerobic exercises (walking, stepper, dancing, VR) Stretching / Tai Chi	Single-component programs
Comparator	Single-component training (“UNI”): a single component (e.g., balance, yoga, stretching, Tai Chi, walking) – Control group: no exercise or maintenance of usual activity	No comparison group
Outcomes	Fall risk–related parameters: Balance: TUG, Mini-BESTest, SEBT, 1-leg stance Strength: CS-30, isokinetics, hand grip Mobility/Function: gait speed (3–10 m), FSST, stair climb Other: BMD, VO₂max, LBM	Studies lacking quantitative measures of function/falls
Study design	Randomized controlled trials with an intervention duration of ≥ 6 weeks	Cohort studies, cross-sectional studies, case–control studies, qualitative studies, case series, case reports, demonstration projects, reviews, and meta-analyses

### 2.3 Classification of interventions

Within this review, we adopted specific criteria for categorizing physical activity programs into multicomponent (MULTI) and single-component (UNI) interventions. Programs were classified as MULTI if they combined at least two distinct components of physical training (e.g., resistance and balance training, aerobic exercises—including movement-based forms such as dance, Tai Chi, or virtual reality training—plus, for example, flexibility exercises or health education). In contrast, interventions were considered UNI when they focused on a single primary type of activity (e.g., balance training only, walking, Tai Chi alone, or stretching exercises only).

In cases involving complex programs that included elements characteristic of multiple modalities (e.g., programs combining Tai Chi with strength and balance training, or mixed balance-cognitive training with virtual reality components), the intervention was classified as MULTI—provided that these components were implemented within a single, cohesive program. Tai Chi was treated as a standalone modality (UNI), despite including balance and strengthening elements, because in the reviewed studies it represented a unified exercise format (i.e., a targeted “mind–body” training with its own distinct methodology). Similarly, walking at a prescribed pace was considered a single-component intervention, whereas programs combining walking with additional exercises were classified as MULTI.

These classification criteria were applied during the study selection phase: only trials comparing the effects of a MULTI training program with a simpler intervention (UNI) or with no intervention (control group) were included. Studies in which all groups received multicomponent training (with no clearly defined UNI or control group) or those incorporating non-exercise components (home environment modifications or supplementation) were excluded to ensure the review focused solely on the effects of physical exercise. While some degree of subjectivity in classification was unavoidable, this was minimized by having two independent authors cross-verify classification decisions. This approach was intended to maintain consistency in comparisons across studies.

The minimum intervention length of 6 weeks was established based on data showing that neuromuscular adaptations in older adults typically occur after approximately 4 weeks (35), and in accordance with WHO guidelines suggesting that shorter interventions exert only limited effects on fall risk (36).

### 2.4 Selection process

Two authors (K. K. and E. KP.) initially agreed upon the procedure for selecting the results obtained from the databases, and the adopted strategy was subsequently discussed with a third author (W. B.). A manual selection of articles was performed, and only publications with full-text availability were considered. Based on the titles, keywords, and abstracts, the retrieved results were screened for eligibility. Full-text articles were obtained for those deemed potentially relevant; if the full text was not accessible, corresponding authors were contacted. Once acquired, the full texts were screened by two additional authors (Ł. R. and T. A.) to determine eligibility for inclusion in the review. Duplicates were removed manually. Any disagreements were resolved through discussion with the other authors and consultations with another author (P. C.).

### 2.5 Data collection process

The data extraction process was conducted by two independent authors (T. A. and W. B.), who initially analyzed a randomly selected sample of studies meeting the inclusion criteria. After individual assessments, the authors met to verify the consistency of the extracted data and to reach a consensus. Any discrepancies were resolved through discussion. Subsequently, the lead researcher (K. K.) extracted data from the remaining eligible studies, and a second author (E. KP.) performed a final accuracy check of all collected in-formation. Data were extracted from the full texts of all studies included in the review.

### 2.6 Data items

The following data were extracted:

- Outcomes: falls (number, % of participants, IRR/RR with 95% CI), balance (TUG, Mini-BESTest, SEBT, 1-leg stance), strength (CS-30, isokinetics, hand grip), mobility (gait speed, FSST, stair climb), other (BMD, LBM, VO₂max, steps/day, adverse events [AE]).
- Other variables: sample size (N), age ± SD, gender, MULTI components (type, dos-age, duration), type of UNI/control group (type, dosage), study design (RCT, ITT, follow-up ≥ 85%).

### 2.7 Effect measures

All included studies reported continuous outcomes (balance, strength, mobility, physiological parameters), and—in one case—a dichotomous outcome (falls). For each intervention and comparison group, we extracted the following data as reported by the authors:

- For continuous outcomes—mean differences (MD) or percentage changes (%*Δ*), along with measures of variability (SD) and *p*-values. When MD or %Δ values were directly re-ported by the authors, they were accepted without additional calculations. If a study re-ported only MD and *p*-values without confidence intervals, this was noted in Table 2.
- For falls (dichotomous outcome)—Incidence Rate Ratios (IRR) with 95% confidence intervals were extracted when available. In one instance where the IRR was reported with-out a CI, only the IRR value and its statistical significance were cited.

**Table 2:** Summary of included publications.

Publication	N; age (mean ± SD)	Characteristics of MULTI interventions	Characteristics of the UNI/CONTROL group	Balance	Strength	Mobility	Duration	Effect measures (95% CI)	Conclusions
Sadeghi et al. 2021 (38)	58 men; 71.8 ± 6.1 years; 100% male	MIX: balance + VR + strength (40 min, 3×/week, 8 weeks)	BT: traditional balance training (40 min, 3×/week, 8 weeks) VR: virtual reality (40 min, 3×/week, 8 weeks) CON: waitlist control	1-leg stance ↑; tandem stance ↑; TUG ↓*	Isokinetic strength of quadriceps/hamstrings ↑*	TUG ↓*; 10 mWT ↑*	8 weeks	MIX vs. BT: ΔTUG ≈ − 1.0 s; MIX vs. VR: ΔTUG ≈ − 0.8 s (*p* < 0.05)	MULTI improved strength, balance, and mobility more than both single-component programs.
Li et al. 2018 (39)	670 (224 TJQMBB, 223 MME, 223 STR); 77.7 ± 5.6 r.; 65 %F	TJQMBB: therapeutic Tai Ji Quan (2 × 60 min/week, 24 weeks) MME: balance + aerobic + strength + flexibility (2 × 60 min/week, 24 weeks)	STR: stretching (2 × 60 min/week, 24 weeks)	-	-	-	24 weeks	Falls IRR: TJQMBB 0.42 vs. STR; MME 0.60 vs. STR; TJQMBB vs. MME: 0.69*	The UNI program (Tai Ji) reduced falls more effectively than MULTI; both were superior to the control group.
Campos et al. 2024 (33)	MG *n* = 50; WG *n* = 41; MG: 69.8 ± 6.4 r.; WG: 67.8 ± 6.1 r.; MG 22% M, WG 7.3% M	Multicomponent – strength + balance + aerobic + stretching; 16 weeks, 2×/week	Walking: 40 min outdoor walking (32 sessions; 2×/week; 16 weeks)	Mini-BEST: MG ↑0.44 points*; WG – 0.05	Handgrip and 5 × STS ↑ in both groups (no differences between them)	Gait speed: WG ↑0.62 s*; MG –	16 weeks	Mini-BESTest: +3.3 points in MULTI (*p* < 0.01) vs. no change in UNI; Gait speed ↑ only in UNI	MULTI improved postural control, UNI improved gait speed; both increased strength.
Zhuang et al. 2014 (40)	50 (MG *n* = 22; CTR *n* = 28); 60–80 years	Strength (squats, core) + balance + 8-form Tai Chi (3×/week, 12 weeks)	CON: no additional exercise (maintenance of usual activity)	SEBT: ↑8–20% in 8 directions*	CS-30 ↑54.7%; TUG ↓17.6%	Gait speed ↑; joint ROM ↑*	12 weeks	TUG −17.6%; CS + 54.7% (*p* < 0.01 vs. control)	MULTI significantly reduced TUG times and improved balance compared to no exercise.
Daly et al. 2020 (41)	162 (81 Osteo-cise; 81 CON); ≥ 60 r.; 76 %F	Osteo-cise: PRT + impact + balance + education + behavioral support (3×/week, 12 months)	CON: no exercise (advised to maintain usual activity)	FSST ↓6%*; stair climb ↓5%*	Muscle strength ↑10–13%*	Sit-to-stand ↑16%*	18 months (12 + 6)	No significant difference in falls; FSST −6%, TSC − 5% (*p* < 0.05)	Long-term MULTI improved function and strength but did not reduce fall risk compared to control.
Kwon et al. 2008 (37)	40; 77.2 ± 2.9	Multicomponent training (20 min stretching, 30 min low-impact aerobic exercise, 30 min resistance training); 3×/week for 24 weeks; progressive aerobic intensity (40–75% HRR).	Control group (no exercise)	Max step length ↑4.1 cm*; Functional reach ↑9.1 cm*	LBM ↑; lower limb muscle strength ↑; trochanteric BMD ↑*	10 m max walking time ↓3.2 s*	24 weeks	VO₂max: +154.2 ml/min (*p* < 0.01); Lower limb muscle mass: +1.2 kg (*p* < 0.05); Trochanteric BMD: +0.02 g/cm^2^ (*p* < 0.05).	Multicomponent training significantly increased VO₂max (+154.2 mL/min), lower limb muscle mass (+1.2 kg), and trochanteric BMD (+0.02 g/cm^2^), improved balance (functional reach +9.1 cm) and gait speed (10 m time ↓3.2 s) in older women. The program effectively reduces fall risk factors compared to no intervention.

*p < 0.05; **p < 0.01; for IRR: *p* < 0.01; N, number of participants; ±SD, mean value ± standard deviation; M, men; F, women; CON / CTR, control group; UNI, single-component intervention; MULTI, multicomponent intervention; VR, virtual reality; BT, traditional balance training; TJQMBB, Tai Ji Quan: Moving for Better Balance; MME, multicomponent exercise; STR, stretching; PRT, progressive resistance training; TUG, Timed Up-and-Go; 10 mWT, 10-meter walk test; SEBT, Star Excursion Balance Test; CS-30, 30-Second Chair Stand Test; FSST, Four-Square Step Test; Stair climb, stair climbing test; BMD, bone mineral density; LBM, lean body mass; VO₂max, maximal oxygen uptake; PAM, physical activity monitor (steps/day); ROM, range of motion; MG, multicomponent group; WG, walking group; ↑, increase; ↓, decrease; BMD, bone mineral density; MET, metabolic equivalent of task; wks, weeks; mth, months.

For studies that did not provide raw data sufficient to calculate missing 95% CIs or SDs, we relied solely on the effect measures published by the authors. All extracted effect sizes are presented in Table 2.

### 2.8 Study risk of bias assessment

The methodological quality of all included studies was assessed by two independent authors (K. K. and E. KP.) using the PEDro scale. The PEDro scale consists of 10 items evaluating aspects such as random allocation, blinding of participants and assessors, completeness of follow-up, and appropriateness of statistical analyses. Any discrepancies in scoring were resolved through discussion, and final decisions were made in consultation with the remaining authors. All included publications were assessed according to the following PEDro criteria:

- Randomization—whether allocation to groups was random.
- Allocation concealment—whether allocation concealment was described and implemented.
- Comparability at baseline—whether groups were comparable in baseline characteristics.
- Patient blinding—whether participants were blinded to group allocation.
- Therapist blinding—whether therapists administering the intervention were blinded.
- Assessor blinding—whether outcome assessors were blinded to group allocation.
- At least 85% follow-up—whether at least 85% of participants completed the study.
- Intention-to-treat analysis—whether an intention-to-treat analysis was conducted.
- Between-group statistical comparisons—whether statistical comparisons between groups were performed.
- Point measures and measures of variability—whether results were reported as mean values with measures of variability (SD or CI).

The maximum possible score was 10 points. Based on the total PEDro scores, each study was classified into one of three methodological quality categories: “high quality” (6–10 points), “moderate quality” (4–5 points), and “low quality” (0–3 points). Final quality scores for all included studies are presented in Table 3.

**Table 3:** PEDro scale for the included studies.

Study	1	2	3	4	5	6	7	8	9	10	Total
1. Kwon et al. (37)	+	−	+	−	−	−	+	−	+	+	5/10
2. Sadeghi et al. (38)	+	−	+	−	−	+	+	−	+	+	6/10
3. Li et al. (39)	+	−	+	−	−	+	+	+	+	+	8/10
4. Zhuang et al. (40)	+	−	+	−	−	−	+	−	+	+	6/10
5. Daly et al. (41)	+	−	+	−	−	−	+	−	+	+	6/10
6. Campos et al. (33)	−	−	+	−	−	−	+	+	+	+	6/10

PEDro items: 1. Randomisation; 2. Allocation concealment; 3. Comparability at baseline; 4. Patient blinding; 5. Therapist blinding; 6. Assessor blinding; 7. At least 85% follow-up; 8. Intention to treat analysis; 9. Between-group statistical comparisons; 10. Point measures and measures of variability. Marks: (+), item fulfilled; (−), item not fulfilled.

## 3 Results

### 3.1 Study selection

**Figure 1:**
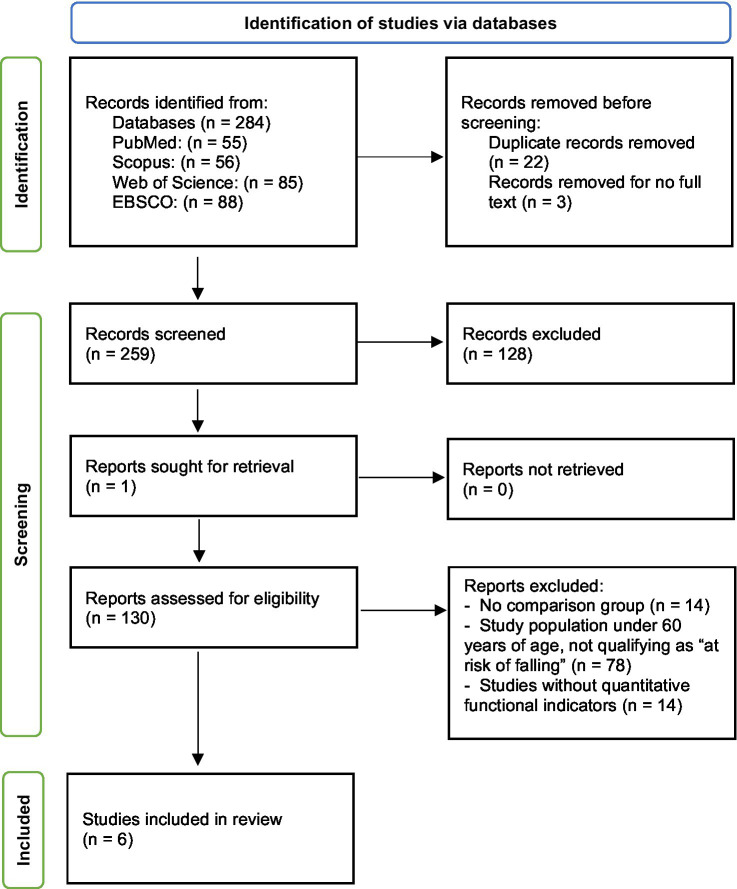
PRISMA flowchart.

A total of 284 records were initially identified (Figure 1). After removing 22 duplicates, the titles and abstracts of 259 articles were screened by two independent authors (K. K. and E. KP.), with only 7.4% (*n* = 19) raising uncertainties regarding the inclusion criteria; all disagreements were resolved through discussion. A total of 130 articles were selected for full-text review, of which 124 were excluded for the following reasons: lack of comparison between a multicomponent and a single-component program (*n* = 14), population aged < 60 years or residing in long-term care facilities (*n* = 78), and absence of functional indicators related to fall risk assessment (*n* = 32). As a result, six randomized con-trolled trials were included in this review (Table 1). Additionally, reference list screening of the included studies and a final search of major databases following the initial selection process identified further records; however, none met the inclusion criteria (e.g., lack of a comparison group or interventions involving fewer than two components).

A meta-analysis was not feasible due to substantial heterogeneity in study designs, intervention protocols, and outcome measures (e.g., varying balance, strength, and mobility tests, as well as non-standardized fall metrics). One author was contacted to request the full version of a publication.

### 3.2 Risk of bias in studies

Only one of the analyzed studies was classified as having “moderate” methodological quality (37). The remaining studies received scores ranging from 6 to 10 points, qualifying them as having “high” methodological quality (33, 38–41). The results are presented in Table 3.

### 3.3 Study characteristics

The review included six randomized controlled trials published between 2008 and 2024, involving a total of 1,071 participants aged 60 to 80 years (mean age 69.8–77.7 ± 2.9–6.4 years) (33, 37–41). The studies recruited both community-dwelling older adults and groups receiving additional educational support (e.g., Osteocise). The proportion of female participants ranged from 0% (38) to 76% (41), with the study by Kwon et al. (37) including only women. Multicomponent interventions (MULTI) consisted of at least two of the following components: resistance strength training, balance exercises, aerobic activities (or dance/Tai Chi/VR), stretching, or educational-behavioral elements. Intervention durations ranged from 8 weeks (3 × 40 min/week) to 18 months (3 × for 12 months + 6-month follow-up). Comparison groups received either a single training component (UNI: traditional balance training, VR, Tai Ji, stretching, or walking) or instructions to maintain usual daily activity (CON). The frequency of training sessions ranged from 2 to 3 times per week, with each session lasting 40–60 min. Outcome measures included balance tests (TUG, Mini-BESTest, SEBT, Functional Reach, one-leg stance), strength assessments (CS-30, isokinetics, hand grip, 5 × STS), mobility tests (gait speed, FSST, stair climb, 10 mWT), as well as physiological and morphometric parameters (VO₂max, LBM, BMD), and fall-related metrics (IRR with 95% CI).

### 3.4 Effect sizes

Effect sizes for continuous data were expressed as standardized mean differences (Cohen’s *d*). The *d* values were approximately calculated based on the difference in mean outcomes between the intervention and comparison groups, divided by the pooled standard deviation. A positive *d* value indicates a better outcome in the MULTI group. For event rate measures (e.g., number of falls), incidence rate ratios (IRR) were used, comparing the rate of falls in the intervention group to that in the control group (IRR < 1 indicates a lower fall rate in the MULTI group). Key findings from the individual studies, including between-group differences and corresponding effect sizes, are presented in Table 4. All estimated effects were interpreted according to standard conventions (0.2, small effect; 0.5, moderate; ≥0.8, large effect).

**Table 4:** Effect sizes of interventions in individual studies.

Study	Primary comparison (Outcome)	Outcome difference (MULTI vs. Comparator)	Effect size
1. Kwon et al. (37)	Multicomponent Training vs. Control (10-Meter Walk Time)	The 10-meter walk time was reduced by approximately 3.2 s in the exercise group compared to the control group.	d = 1.5 (large effect)
2. Sadeghi et al. (38)	Combined Training (Balance Training + Virtual Reality) vs. Traditional Balance (TUG)	TUG time was approximately 1 s shorter in the MIX group compared to traditional balance training.	d = 0.8 (large effect)
3. Li et al. (39)	Tai Chi vs. Stretching (Fall Rate)	There were 58% fewer falls in the Tai Chi group (152 vs. 363 falls) compared to the control group.	IRR = 0.42 (large effect)
4. Zhuang et al. (40)	Combined Training (Strength + Balance + Tai Chi) vs. Control (TUG)	TUG time decreased by 17.6% in the exercise group, with no change observed in the control group.	d > 1.0 (large effect)
5. Daly et al. (41)	Osteo-cise Program vs. Control (Stair-Climbing Time)	Stair-climbing time decreased by 5% in the exercise group, with no change observed in the control group.	d = 0.5 (moderate effect)
6. Campos et al. (33)	Multicomponent Training vs. Walking (Mini-BESTest Outcome)	Mini-BESTest score increased by 3.3 points in the MULTI group, with no improvement observed after walking-only intervention.	d = 0.8 (large effect)

BT, Balance Training; VR, Virtual Reality; TUG, Timed Up and Go Test; Mini-BESTest, Mini Balance Evaluation Systems Test; IRR, Incidence Rate Ratio (fall frequency ratio).

Due to the heterogeneity of outcome measures (different tests and units), a formal meta-analysis was not conducted—values for I^2^ and τ^2^ were not calculated. Instead, heterogeneity was assessed qualitatively, taking into account differences in study populations, intervention duration, type, and exercise intensity.

### 3.5 Effect of the intervention on balance

All MULTI programs demonstrated significant improvements in balance measures compared to single-component interventions or control groups. In the study by Sadeghi et al., improvements were noted in single-leg and tandem stance times, along with a reduction in TUG time by approximately 1 s (*p* < 0.05) in the mixed group (balance + VR + strength) compared to traditional balance training and VR alone (38). Zhuang et al. observed an 8–20% increase in SEBT scores and a 17.6% reduction in TUG times (*p* < 0.01) compared to a no-exercise control group (40). Campos et al. reported a 0.44-point improvement in the Mini-BESTest in the MULTI group, while the walking group showed no change (33).

### 3.6 Effect of the intervention on muscle strength

Multicomponent programs significantly increased lower limb muscle strength and hand grip strength. Sadeghi et al. reported improvements in isokinetic strength of the quadriceps and hamstrings (*p* < 0.05), as well as enhanced 10 mWT performance in the MIX group compared to balance training or VR alone (38). Zhuang et al. observed a + 54.7% increase in CS-30 scores (*p* < 0.01), while Campos et al. found significant improvements in hand grip strength and 5 × STS performance in both groups, with no significant differences between them (33, 40). In the study by Kwon et al., the program led to an increase in LBM and lower limb strength (±1.2 kg, *p* < 0.05) (37).

### 3.7 Effect of the intervention on mobility

MULTI improved gait speed and mobility. Sadeghi et al. reported a reduction in TUG time and improvement in the 10 mWT (*p* < 0.05) (38). Zhuang et al. demonstrated an in-crease in gait speed and joint range of motion (*p* < 0.05) (40). Daly et al. observed a 6% de-crease in FSST time and a 5% reduction in stair-climb times after 18 months of MULTI (*p* < 0.05) (41).

### 3.8 Effect of the intervention on other physiological and structural parameters

Multicomponent programs positively affected aerobic capacity, bone mineral density, and lean body mass. Kwon et al. reported an increase in VO₂max by 154.2 mL/min (*p* < 0.01), lower limb LBM by 1.2 kg (*p* < 0.05), and trochanteric BMD by 0.02 g/cm^2^ (*p* < 0.05) after 24 weeks of resistance training, aerobic exercise, and stretching (37).

### 3.9 Effect of the intervention on fall risk

A reduction in fall incidence (IRR) was reported in two studies. Li et al. showed that the Tai Ji Quan program (UNI) reduced the IRR to 0.42 compared to stretching, while MME (MULTI) reduced it to 0.60 versus stretching; the difference between UNI and MULTI was IRR = 0.69 (*p* < 0.05) (39). Daly et al. did not observe a significant difference in fall incidence between the MULTI and control groups after 18 months of intervention (41).

### 3.10 Effectiveness of multicomponent programs vs. single-component training

Multicomponent programs (MULTI), combining resistance training, balance exercises, and aerobic activity, generally demonstrated greater benefits in improving balance and muscle strength compared to single-component protocols (UNI). This was reflected, for instance, in a significant reduction in TUG time by approximately 1 s, increases in SEBT scores by 8–20%, and greater isokinetic quadriceps strength (*p* < 0.05) in MULTI groups versus balance or VR-based training. However, in simpler functional tests such as hand grip or 5 × STS, moderate UNI programs (e.g., walking) yielded comparable outcomes. While MULTI programs in some studies also improved gait speed and aerobic capacity (VO₂max +154 mL/min, lean body mass +1.2 kg, BMD + 0.02 g/cm^2^), walking-focused interventions proved equally or even more effective in optimizing gait speed. Similarly, Tai Ji Quan training was more effective in reducing fall risk (IRR 0.42) than multicomponent interventions (IRR 0.60).

Sadeghi et al. and part of the results reported by Daly et al. presented outcomes as *F*-values and η^2^ without providing raw data tables; the journal editors did not include these data in the main text or in the supplementary materials available in PDF format—therefore, these rows are marked as “no means reported” (38, 41). The available raw data indicate an advantage of multicomponent training in improving key functional markers (TUG, CS-30, Mini-BESTest). At the same time, the reporting gap (absence of means and standard deviations in three out of six studies) limits the feasibility of full quantitative synthesis and highlights the need for standardization in outcome reporting in geriatric physiotherapy research on fall prevention (Table 5).

**Table 5:** Raw values in individual functional tests.

Author, year (*N* = Intervention / Comparator)	Scale / test (unit)	Raw means ± SD (Baseline → Follow-up)	% Change (INT vs. CTRL)
Zhuang 2014 (40) (22 / 28)	CS-30 (reps / 30 s)	17.5 ± 4.7 → 27.1 ±? †	+55% vs. 0%
	TUG (s)	8.4 ± 2.0 → 6.9 ±? †	−17.6% vs. + 3.8%
	Functional Reach (cm)	30.8 ± 5.1 → 32.3 ±? †	+4.8% vs. − 1.6%
	SEBT (8 Directions)	eg. Anterior 58.6 ± 6.8 → 69.6 ± 6.8	+19–41% (depending on direction)
	Gait speed (m/s)	1.10 ± 0.18 → 1.29 ± 0.22	+17.3% vs. + 0.9%
Kwon 2008 (37) (20 / 20)	Max-step length (cm)	49.9 ± 6.9 → 54.0 ± 6.2	+8.2% vs. − 1.8%
	Functional Reach (cm)	13.3 ± 4.0 → 22.4 ± 3.5	+68% vs. − 5.5%
	10 m-walk (s)	11.2 ± 2.3 → 8.0 ± 1.4	−28.6% vs. + 52%
Campos 2024 (33) (50 / 41)	Mini-BESTest (points)	5.16 ± 0.71 → 5.60 ± 0.57	+8.5% vs. − 0.9%
	Handgrip (kg)	23.1 ± 6.5 → 25.3 ± 6.0	+9.5% (oba programy)
	5 × STS (s)	11.4 ± 3.1 → 10.0 ± 2.6	−12.3% (oba programy)
	3 m gait speed (m/s)	0.92 ± 0.17 → 0.93 ± 0.15	+1.1% vs. + 10.4%*
	Sit-and-Reach (cm)	22.8 ± 6.4 → 22.5 ± 6.5	−1.3% vs. + 13.6%*
Daly 2020 (41) (81 / 81)	Four-Square Step Test (s)	9.0 → 8.5	−6% (CTRL 0%)
	Stair-climb (s)	16.0 → 15.2	−5% (CTRL 0%)
	30 s Sit-to-Stand (rep)	10.6 → 12.3	+16% (CTRL 0%)
Sadeghi 2021 (38) (14 / 14/ 15/ 15)	Single-leg stance (firm, s)	No Means Reported †	–
	Tandem stance (s)	No Means Reported †	–
	TUG (s)	Values Not Reported †	–
	10 m Walk Test (m/s)	Values Not Reported †	–

†Only baseline values ± SD and mean change ± SD were reported in the article; the percentage change was derived from these values, while the SD for the final value was not provided.

## 4 Discussion

### 4.1 Importance of training effects on balance

In the analyzed studies, a significant improvement was observed in balance abilities was observed in older adults who participated in training programs, especially those of a multicomponent nature. Interventions combining various types of exercises led to significant improvements in balance test results compared to both control groups and single-component interventions (33, 38, 40). For example, in the study by Sadeghi et al., an 8-week combined program—involving balance training, strength training, and virtual reality exercises—significantly increased the time participants could stand on one leg and improved stability in the tandem position (38). At the same time, a reduction of approximately 1 s in the Timed Up and Go (TUG) test was noted compared to groups performing only balance training or VR training. Similarly, Zhuang et al. observed significant improvements in dynamic balance—SEBT scores increased by 8–20%, and TUG time was reduced by nearly 18%—after applying a complex exercise program combining strength and balance elements, while no such changes occurred in the control group (40). The study by de Campos et al. also confirms the superiority of the multicomponent approach: individuals in the multicomponent training group showed a small but statistically significant improvement in the mini-BESTest postural balance test, while the group performing only regular walking (as a single-component training) did not show significant changes in this measure (33). Increased time standing on one leg or better results in dynamic stability tests translate into greater confidence in maintaining posture during daily activities, such as walking on un-even surfaces or rising from a chair. All of the mentioned interventions, by strengthening postural control mechanisms, potentially contribute to reducing the risk of tripping and falling in older adults. It should be noted that differences in the balance tests used (from static to dynamic) do not change the overall conclusion: appropriately selected training—especially one containing balance exercises—effectively improves balance functions regardless of the specific assessment method used (38, 40).

### 4.2 Effect of the intervention on muscle strength

The increase in muscle strength—particularly in the lower limbs—is one of the most important outcomes observed in the exercise programs studied (42, 43). All interventions involving resistance training or general conditioning led to significant strength gains compared to no exercise, and often also outperformed single-component interventions in this regard (33, 38, 40). Sadeghi et al. demonstrated that combining balance training with strength exercises (along with VR elements) resulted in a significant increase in maximum quadriceps and hamstrings strength measured isokinetically in older men—this effect was more pronounced than that of balance training or VR training alone (38). Similarly, Zhuang et al. observed an improvement in functional lower limb strength: the number of repetitions in the 30s Chair Stand Test increased by over 50% from baseline in participants who took part in the integrated exercise program, while no such change was observed in the control group (40). These results indicate that even relatively short-term interventions can significantly counteract sarcopenia and muscle weakness in older adults.

In some studies, even simple forms of physical activity showed the ability to improve strength, though to a more limited extent. In the experiment by de Campos et al., both the multicomponent program and the single-component walking-based program led to increased hand grip strength and a reduction in the time taken to complete the 5 × STS test—and importantly, the differences between these groups were statistically insignificant. This suggests that even moderate, steady aerobic activity (regular walking) can offer some benefits in functional strength, likely by engaging postural and lower limb muscles during daily movement (33). However, when the goal is to maximize muscle strength gains, multi-component interventions that include dedicated resistance training seem more effective. For example, Kwon et al. showed that a comprehensive 24-week program combining aerobic, strength, and stretching exercises in older women led to an average increase of over 1 kg in lean body mass, which typically correlates with increased muscle strength and indicates muscle mass growth (37).

From a clinical perspective, the strength gains achieved translate into improved ability to perform daily activities, such as standing up from a chair, climbing stairs, or lifting groceries (44). Moreover, increased muscle strength may improve functional reserve in patients—even if the strength gain seems modest (a few percent), for an older person, this can make the difference between being able to perform a task independently or needing assistance (31). In conclusion, the evidence from the reviewed studies clearly indicates that incorporating resistance training (preferably as part of a multicomponent program) is justified and effective in the older adult population, helping to counteract age-related muscle strength loss (37, 38, 40).

### 4.3 Changes in mobility

Multicomponent exercise programs generally contributed to increased gait speed, agility, and overall locomotor function in participants, which was reflected in the results of various field tests (38, 40, 41). For example, Sadeghi et al. reported that the group under-going the combined training program achieved better results in the TUG test (faster completion of the task of standing up, walking, and returning to the chair) compared to the groups doing more narrow-range exercises (38). Furthermore, only the multicomponent group showed a improvement in gait speed over a 10-meter distance, suggesting that combining balance and strength exercises translated into better movement efficiency over short distances. In Zhuang et al., an intervention including strength and endurance training led to a significant increase in average gait speed and an increase in the range of motion in lower limb joints, while the inactive control group showed no such changes (40).

It is worth noting that the beneficial changes in mobility included not only simple locomotor tasks but also more complex activities requiring coordination and agility. Daly et al. demonstrated that after 12 months of supervised multicomponent training (followed by 6 months of home-based training), older adults showed improved ability to navigate obstacles and change direction—the time to complete the Four Square Step Test decreased by about 6%, and stair climb time improved by 5% compared to baseline (41). Although these changes may seem small in percentage terms, for older adults, they could mean easier and safer navigation of architectural obstacles in both home and outside environments (45).

In terms of comparing different training approaches, an interesting result came from the study by de Campos et al. (33). It turned out that a walking-focused program (regular walking) was just as effective, and in some aspects even more effective, in improving locomotor parameters than the multicomponent program. Participants in the walking pro-gram showed a comparable increase in gait speed to the multicomponent group, suggesting the principle of training specificity—practicing a specific activity (walking) most effectively improves that skill. Meanwhile, the multicomponent program provided broader benefits for overall fitness, but did not surpass the walking program in terms of increasing step speed (33).

From a clinical perspective, improving mobility means greater independence and quality of life for older adults. Even a small increase in gait speed (a few centimeters per second) can shift an individual from the risk of losing independence to a safer, independent mobility status within the community. A 1-s reduction in TUG time, observed in some interventions (38, 40), can be interpreted as an improvement in the ability to quickly stand up and start walking—a crucial action, for example, in responding to fall threats. Of course, it is important to note that the size of the training effect depended on the type of exercise and control group: where the reference point was inactivity (40), the mobility improvements were more pronounced, while when compared to active control training, differences between groups became less clear.

### 4.4 Effectiveness of tai chi—possible mechanisms underlying functional improvement

The findings of this review suggest that Tai Chi training may be effective in improving balance and preventing falls among older adults. In the study by Li et al., the Tai Chi group experienced a 31% lower fall rate compared to the group performing multicomponent exercises (39). Regular practice of Tai Chi enhances sensorimotor integration—participants learn to use proprioceptive and visual cues more effectively to maintain balance (46). Neurophysiological research suggests that Tai Chi may induce neuroplasticity in brain regions responsible for postural control and balance responses. For instance, improvements have been observed in neural networks involved in maintaining equilibrium and in the complexity of postural dynamics following extended Tai Chi practice (46).

Moreover, the slow, precise movements characteristic of Tai Chi require constant postural adjustments and shifts in the center of mass, thereby training reactive balance responses to perturbations. Gatts and Woollacott demonstrated that even a few weeks of Tai Chi training significantly reduced the latency of reflexive lower limb muscle responses to sudden balance disturbances (e.g., tripping) in older adults (47). In other words, Tai Chi may enhance the ability to react more quickly and appropriately to slips or trips—directly reducing fall risk. It is worth emphasizing that Tai Chi primarily focuses on dynamic balance, coordination, and corrective reaction training, which may provide an advantage over multicomponent programs that, although comprehensive, may not emphasize balance training to the same extent.

In summary, the mechanisms underlying the effectiveness of Tai Chi likely include neuromotor adaptations (e.g., improved reaction time, coordination of antagonistic muscles), increased cognitive reserve and attentional focus (through mindfulness), and improved integration of sensory information critical for maintaining balance (46, 47). These features may be less developed in multicomponent programs, making Tai Chi a valuable component or alternative in fall prevention strategies.

### 4.5 Physiological and structural variables—clinical significance

Some of the analyzed studies went beyond standard functional measures, assessing the impact of exercise on physiological variables (such as aerobic capacity) and structural variables (such as bone density or muscle mass). The results of these studies suggest that physical interventions can induce beneficial adaptations at the level of the cardiovascular-respiratory and musculoskeletal systems, which have significant clinical implications for the health of older adults (48). Kwon et al. in their study showed that a 6-month multi-component training program in older women resulted in an average increase of 154 mL/min in maximal aerobic capacity (VO₂max) (37). This improvement indicates enhanced cardiovascular function, which practically translates to increased exercise tolerance—seniors can engage in moderate activity for longer periods without excessive fatigue (49). This is beneficial not only for the training itself (allowing gradual increases in exercise intensity) but also for daily functioning—better endurance makes it easier to take longer walks or climb more stairs without resting (50).

Physical interventions also affected body composition parameters and selected structural markers of the skeleton. The aforementioned study by Kwon et al. noted, in addition to an increase in lean body mass, a small but significant increase in bone mineral density (BMD) in the femoral trochanter area—an average of 0.02 g/cm^2^ after the exercise program (37). Although the absolute change in density seems small, in older adults, even halting the decline in BMD or a minimal increase is a desired outcome, potentially leading to a reduced risk of osteoporosis and osteoporotic fractures in the long term (51). Daly et al. focused on the structural aspects and demonstrated that a 12-month intensive multicomponent program (including bone-loading exercises), followed by 6 months of maintenance activity, significantly impacted bone parameters (41). Participants in the multicomponent training group showed maintenance or improvement in BMD in critical areas (e.g., regions important for hip strength), as well as beneficial changes in bone tissue microarchitecture, compared to the control group that did not undergo training. These results suggest that regular physical activity can serve as “osteoprotective” for older adults, complementing or enhancing standard osteoporosis prevention interventions (e.g., calcium and vita-min D supplementation or pharmacotherapy).

One of the studies also assessed flexibility and range of motion as complementary indicators of physical fitness. de Campos et al. reported that only the multicomponent program, which included varied forms of exercise (likely with stretching elements), led to a clear improvement in flexibility in participants, whereas the single-component walking program did not significantly affect this parameter (33). Increased muscle flexibility and joint range of motion in older adults can facilitate the performance of daily activities (e.g., reaching for objects, dressing) and reduce the risk of injuries resulting from mobility limitations (52).

In conclusion, although not all of the analyzed studies thoroughly examined physio-logical and structural variables, those that did indicate multidimensional benefits associated with participation in exercise programs. Improvements in aerobic capacity, muscle mass gain, or even partial inhibition of bone density loss constitute important complements to improvements in functional parameters (53). From a clinical perspective, this translates to an overall improvement in health—seniors become not only more physically fit but also more resistant to internal risk factors (e.g., osteoporosis, frailty syndrome). However, it should be noted that achieving such adaptations requires appropriately intense and long-term training, aimed at specific physiological goals (e.g., cardiovascular load exercises to improve VO₂max, or resistance exercises to stimulate bone tissue) (54–56). Differences in intervention protocols across studies (duration, intensity, presence of aerobic or strength components) may explain discrepancies in the magnitude of observed effects. Nonetheless, the results clearly support the inclusion of diverse training elements in programs for older adults—not only for improving their current fitness but also for long-term cardiovascular, muscular, and bone health.

### 4.6 Reduction of fall risk—effectiveness analysis

The key goal of physical interventions in older adults is fall prevention, which has serious health consequences. Among the six studies included, only two directly assessed the incidence of actual falls as an outcome of the intervention, which reflects the logistical challenges (the need for long-term observation of large groups) associated with such measurements. The results of these two randomized controlled trials are inconclusive. In a large clinical trial by Li et al., involving over 600 older adults at high fall risk, classical multicomponent training was compared to a specialized intervention focused on balance (therapeutic Tai Ji Quan) (39). The results clearly favored the balance-focused training: during the study, the Tai Chi group experienced 58% fewer falls than the control group (Incidence Rate Ratio IRR ~ 0.42 compared to control) (39). The group undergoing the multicomponent program also showed a lower fall incidence compared to the control group, though the risk reduction was less dramatic (IRR ~ 0.60, about 40% fewer falls than the control group). Notably, Tai Ji Quan was significantly more effective than the multicomponent intervention, with a difference of about 31% in favor of Tai Chi (39). This strong effect is at-tributed to the specificity of Tai Ji exercises, which focus on dynamic balance, coordination, and corrective reaction training, directly enhancing the ability to prevent balance loss in threatening situations. The results of this study provide evidence that an appropriately selected exercise program, particularly one focused on improving balance, can significantly reduce fall incidence in a high-risk senior population.

A very different picture emerges from the study by Daly et al., which also monitored actual falls but in a slightly different population and context (41). In this 18-month experiment, the focus was on older adults participating in a multicomponent exercise program, with the primary goal of improving bone density and physical function, not specifically fall prevention. Despite achieving the previously mentioned benefits in fitness parameters, the authors found no statistically significant difference in fall incidence between the exercise and control groups at the end of the study (41). In other words, the overall fitness program—while beneficial for strength, balance, and mobility—did not automatically translate into a reduction in fall numbers in the sample. There are several possible explanations for this result. First, participants in Daly et al. may not have belonged to a very high fall-risk group at the start of the intervention (e.g., they were relatively fit seniors, with low fall rates even without training) (41). In such a population, demonstrating additional fall reduction is statistically more difficult. Second, the effectiveness of the program in fall prevention may depend on the specificity of the exercises used—it is possible that, although the program was multicomponent, it did not emphasize balance or reactive exercises sufficiently (focusing more on strength and endurance components for bone health), which may have prevented it from developing adequate reserves for fall prevention in participants (32, 57). Additionally, the second phase of the study (6-month transition to independent activity) may have involved a reduction in exercise intensity or frequency, weakening the long-term protective effect. These factors may explain why the final number of falls in the intervention group did not differ from the control group, despite initial functional improvements.

It is worth noting that the other four studies in the review did not directly report fall incidence, primarily due to the short duration of the interventions (e.g., 8 weeks in Sadeghi et al. or 12 weeks in Zhuang et al.) and limited sample sizes, which prevented fall analysis. In such cases, a reduction in fall risk can only be inferred indirectly, based on improvements in risk factors (balance, strength, and gait). Indeed, considering the significant improvements in these functional domains in most studies, it can be expected that properly conducted exercises will eventually lead to a lower susceptibility to falls. However, the lack of direct fall data in short-term RCTs reminds us that longer, more comprehensive studies are necessary to assess the actual effectiveness of fall prevention.

In conclusion, evidence from the analyzed studies suggests that exercise programs can reduce the incidence of falls in older adults, but this effectiveness is greatest when the training includes a strong balance component and is applied to individuals genuinely at risk of falling (39). General fitness programs also have the potential to improve safety, but they may not always be sufficient to reduce fall numbers, especially if they do not provide a specific stimulus for improving protective reflexes against falls (41). For clinical practice, this means that to effectively prevent falls, physical activity should not only be recommended but also include exercises specifically focused on dynamic balance, coordination, and rapid response to instability.

### 4.7 Sustainability of effects, adherence, and environmental factors

When analyzing the effectiveness of physical interventions in older adults, it is essential to consider the sustainability of the achieved effects and the level of adherence to exercise recommendations. Numerous studies indicate that the benefits of exercise may diminish after the completion of supervised programs if participants fail to maintain an adequate level of physical activity. Indeed, adherence (i.e., regular participation in training) presents a challenge: in the long-term study by Daly et al., the average attendance rate was only 59% at 12 months, and it dropped to 45% during the subsequent 6 months (41). Similarly, global statistics show that the proportion of older adults meeting physical activity recommendations is low and decreases with age (in the United States, only about 15% of people aged 65 and over achieve the recommended 150 min of weekly physical activity) (58). This suggests that maintaining motivation and consistency in exercise outside the research context is difficult, and without it, long-term functional improvements are unlikely to persist. In the reviewed studies, the follow-up period after the intervention was not always assessed; however, it can be assumed that the rate of continued exercise had a substantial impact on long-term outcomes.

Factors that promote better adherence include social support and an exercise-friendly environment. As demonstrated in systematic reviews, older adults are more likely to remain active if they receive encouragement and assistance from family members or peers (59). Group-based exercise programs—such as organized Tai Chi classes or balance training sessions in senior centers—not only provide health benefits but also foster social integration. Participants motivate each other, which increases attendance and persistence. Interviews with participants indicate that a sense of community and mutual accountability within the group serve as strong motivators for regular participation (58, 59). Equally important are individual factors, such as awareness of the benefits of exercise (i.e., education about its impact on health), self-efficacy, and intrinsic motivation to improve physical fitness (58). None of the included trials reported serious adverse events attributable to the exercise interventions, indicating that both multicomponent and single-component programs were generally safe for older participants.

Successful programs often incorporate components that support these aspects—motivational coaching, progress tracking, gradual intensity progression to prevent discouragement, and involvement of close relatives (e.g., through joint home-based exercises). Environmental factors also play a significant role: access to safe spaces for physical activity (walking paths, senior centers) and the absence of transportation barriers increase the likelihood that older adults will remain active after the conclusion of the study program. In summary, the sustainability of intervention effects depends on continued physical activity, which in turn is influenced by both individual motivation and health status, as well as social support and an environment conducive to an active lifestyle. In the context of older adults, this implies the need for additional measures: education, support group development, and the design of programs that are appealing, tailored to individual abilities, and easily accessible. Such strategies can help transform short-term functional improvements into long-term health benefits.

### 4.8 Multicomponent vs. single-component programs—practical implications

A comparative analysis of multicomponent (MULTI) and single-component (UNI) interventions provides valuable insights for planning exercise and rehabilitation pro-grams for older adults. The gathered data indicate that multicomponent programs—combining different types of exercise such as strength, balance, and aerobic training—offer a broader spectrum of functional benefits. These programs have been shown to be superior to more narrowly focused interventions in terms of improving key motor abilities, especially balance and muscle strength (33, 38, 40). For example, in the study by Sadeghi et al., the group undergoing the integrated program (which included balance elements, virtual reality, and resistance exercises) achieved better results in both balance tests and strength measurements compared to the groups doing only one type of training (38). This means that the synergy of various training stimuli enhances adaptations—resistance training strengthens muscles, which facilitates balance tasks, while balance training, supplemented with strength or aerobic components, can more effectively improve motor functions than isolated balance exercises. As a result, older adults participating in multicomponent programs typically gain better stability, greater strength, and endurance, often with improvements in other health aspects (such as cardiovascular fitness and flexibility), making them more physically capable in an overall sense.

However, this does not mean that single-component training is without value—quite the opposite, some focused forms can be equally effective, and sometimes even more effective, for achieving specific goals. As shown by the results of Li et al.’s study, a specialized Tai Ji Quan program focused on balance significantly outperformed the more general multicomponent program in reducing falls (39). This suggests that if the priority of the intervention is a specific domain (in this case, fall prevention through improved balance), very intense focus on that particular domain may yield the best results. Similarly, de Campos et al. showed that a single-component walking program improved gait speed to the same extent as the multicomponent program—which makes sense, considering that walking training directly targets the ability being measured (33). In this context, single-component interventions are characterized by high specificity of adaptation: they primarily improve the function they target. From a practical point of view, this means that when choosing the type of program for a given patient, their most significant needs should be taken into ac-count. If an individual’s primary issue is slow gait and low endurance, a simple walking-based intervention may bring measurable improvements in these parameters in a short time (33). On the other hand, for a patient with significant balance impairments and a history of falls, the priority should be intense balance training—perhaps even at the expense of other components—because balance is the weakest link determining their safety (39).

Despite the above-mentioned specific benefits, single-component programs have a limited scope of influence—they usually do not significantly affect domains other than the one they are focused on. For example, aerobic training alone (walking) may not significantly improve balance or strength, as confirmed by the lack of changes in the balance test in the walking group in de Campos et al.’s study (33). Therefore, from a long-term and population-level perspective, most geriatric guidelines recommend a comprehensive approach. Our review of six RCTs supports this recommendation: the greatest overall bene-fits were observed in individuals participating in multicomponent programs, and any advantages of targeted interventions appeared mainly in specific, narrow indicators. In clinical practice, this implies that the safest and most effective strategy for older patients is to include both balance-improving elements and exercises that enhance muscle strength and endurance in the training plan. Such a balanced program addresses multiple fall risk factors simultaneously: it strengthens muscles (providing better strength reserves in case of a stumble), trains postural responses (preventing falls during instability), and improves fitness (allowing continued activity without excessive fatigue, which could lead to neglecting safety).

From the perspective of intervention designers and therapists, it is also important to consider patients’ preferences and capabilities. While multicomponent programs are effective, they can be more complex organizationally—requiring access to equipment (e.g., resistance training devices), greater expertise from the instructor (knowledge of different training forms), and participant motivation to perform varied exercises. Some older adults may prefer simpler, more homogeneous forms of activity (e.g., daily walks, Tai Chi classes in peer groups) due to their accessibility and social nature.

### 4.9 Limitations of the study

The comparative analysis of the studies also revealed several limitations. First, the concept of “multicomponent” was not consistently defined across studies—the content of multicomponent programs varied. For example, the intervention described by Li et al. included elements of strength, balance, and aerobic training, but may not have been as intensive in balance training as a specialized Tai Chi program. This could explain why its effect on fall prevention was comparatively smaller (39). On the other hand, the program by Sadeghi et al. included an innovative VR component in addition to conventional exercises. Although its effectiveness in improving function was confirmed, it remains unclear whether the addition of VR was necessary—similar outcomes might have been achieved using standard training alone, raising questions about the cost-effectiveness and real-world feasibility of implementing such technologies (38). Moreover, the small number of included studies (*N* = 6) limits the ability to draw definitive quantitative conclusions. The lack of a meta-analysis—due to the heterogeneity of outcomes and interventions—means that the review relies on narrative synthesis, which by definition offers a lower level of evidence.

Second, in studies that employed active control groups (e.g., walking in de Campos et al. or stretching in Li et al.), the between-group differences were smaller than in studies with entirely passive control groups (40). In practice, this suggests that even basic physical activity provides certain benefits. However, to achieve above-average improvements and full functional gains, more diverse training programs appear necessary.

Third, the duration and sustainability of effects remain a challenge: the benefits achieved through short-term interventions may diminish without continued exercise. Programs like the one in Daly et al. attempted to address this by implementing a “transition to practice” phase, but its limited success suggests that patients require ongoing support and motivation to maintain an adequate level of activity (41). Therefore, clinicians should plan not only the initial training intervention but also long-term strategies for engaging older adults in physical activity.

Furthermore, several studies were assessed as having a moderate risk of bias—blinding of participants to the type of intervention was not always possible, potentially affecting reported functional outcomes. In addition, population characteristics (e.g., age 60 vs. 80 years; exclusively male vs. predominantly female samples) and intervention settings (laboratory vs. community-based) introduced further variability. These limitations collectively suggest caution in generalizing the results: the findings of this review are most applicable to populations similar to those studied (active, independently living older adults) and to interventions with comparable characteristics. Comparable effects cannot be guaranteed in populations with poorer health status or with substantially different program structures.

Despite these limitations, the review offers valuable insights into the effectiveness trends of different forms of physical training. However, the conclusions should be interpreted with appropriate caution. Further research—especially large, randomized trials directly comparing MULTI and UNI interventions—is needed to confirm the observed patterns and enable a full statistical analysis, including an assessment of heterogeneity.

## 5 Conclusion

Both the literature and the results of the reviewed studies indicate that the most com-prehensive improvement in the functional and health status of older adults is provided by multicomponent programs. However, their effectiveness in a specific population depends on the proper selection and intensity of the components. Although MULTI programs demonstrated broader effects on functional parameters, the Tai Ji Quan (UNI) intervention achieved a greater reduction in actual falls (IRR 0.42 vs. 0.60). Therefore, final recommendations should take into account the specific characteristics of the target population, individual preferences, and available resources. Single-component training can successfully serve as an alternative when a full program is not feasible—especially if focused on the most critical deficit of a given individual. For practice, it is crucial to take a holistic approach to the needs of the senior: assessing their fall risk, fitness level, comorbidities, and preferences, and then planning a physical intervention optimal in terms of scope and intensity. This personalized approach, based on established principles of multimodal training, offers the best chance to improve function, reduce fall risk, and maintain these effects through high acceptance and long-term patient participation in the program.

### 5.1 Ethical aspects

The authors of this manuscript were not professionally or academically involved in the development or implementation of any of the evaluated intervention programs. This means that none of the authors participated in the creation of Tai Chi training, multicomponent exercise programs, or other interventions analyzed in this review. We provide this information in the interest of transparency. All interventions were evaluated independently, solely based on data published in the original studies. We also note that no other conflicts of interest have been reported; the authors declare no direct financial benefits or affiliations that could influence the objectivity of the presented results.

## Data Availability

Publicly available datasets were analyzed in this study. This data can be found at: CRD420251045931.
